# Platelet endothelial cell adhesion molecule-1 regulates collagen-stimulated platelet function by modulating the association of phosphatidylinositol 3-kinase with Grb-2-associated binding protein-1 and linker for activation of T cells

**DOI:** 10.1111/j.1538-7836.2010.04025.x

**Published:** 2010-11

**Authors:** L A Moraes, N E Barrett, C I Jones, L M Holbrook, M Spyridon, T Sage, D K Newman, J M Gibbins

**Affiliations:** * Institute for Cardiovascular & Metabolic Research, School of Biological Sciences, University of ReadingReading, UK; † Blood Research Institute, Blood Center of Wisconsin, Medical College of WisconsinMilwaukee, WI, USA; ‡ Blood Transfusion Research Group, King Saud UniversityRiyadh, Saudi Arabia

**Keywords:** GPVI, inhibitory, ITIM, PECAM-1, signaling

## Abstract

*Background:* Platelet activation by collagen depends on signals transduced by the glycoprotein (GP)VI–Fc receptor (FcR)γ-chain collagen receptor complex, which involves recruitment of phosphatidylinositol 3-kinase (PI3K) to phosphorylated tyrosines in the linker for activation of T cells (LAT). An interaction between the p85 regulatory subunit of PI3K and the scaffolding molecule Grb-2-associated binding protein-1 (Gab1), which is regulated by binding of the Src homology 2 domain-containing protein tyrosine phosphatase-2 (SHP-2) to Gab1, has been shown in other cell types to sustain PI3K activity to elicit cellular responses. Platelet endothelial cell adhesion molecule-1 (PECAM-1) functions as a negative regulator of platelet reactivity and thrombosis, at least in part by inhibiting GPVI–FcRγ-chain signaling via recruitment of SHP-2 to phosphorylated immunoreceptor tyrosine-based inhibitory motifs in PECAM-1. *Objective:* To investigate the possibility that PECAM-1 regulates the formation of the Gab1–p85 signaling complexes, and the potential effect of such interactions on GPVI-mediated platelet activation in platelets. *Methods:* The ability of PECAM-1 signaling to modulate the LAT signalosome was investigated with immunoblotting assays on human platelets and knockout mouse platelets. *Results:* PECAM-1-associated SHP-2 in collagen-stimulated platelets binds to p85, which results in diminished levels of association with both Gab1 and LAT and reduced collagen-stimulated PI3K signaling. We therefore propose that PECAM-1-mediated inhibition of GPVI-dependent platelet responses result, at least in part, from recruitment of SHP-2–p85 complexes to tyrosine-phosphorylated PECAM-1, which diminishes the association of PI3K with activatory signaling molecules, such as Gab1 and LAT.

## Introduction

Platelet endothelial cell adhesion molecule-1 (PECAM-1, CD31) is a 130-kDa membrane-spanning glycoprotein (GP) that belongs to the immunoglobulin (Ig) family of cell adhesion molecules and consists of a 574-residue extracellular domain composed of six Ig-like homology domains, a 19-residue transmembrane domain, and an 118-residue cytoplasmic tail [1–3]. PECAM-1 is expressed on the surfaces of endothelial cells and several hematopoietic cell types, including platelets, megakaryocytes, monocytes, neutrophils, natural killer cells, and naïve subsets of T and B cells [4,5].

PECAM-1 is a signaling molecule that plays diverse roles in vascular biology, including modulation of platelet function [6–9], angiogenesis [10], vasculogenesis [11], integrin regulation [12,13], T-cell and B-cell activation [14,15], and mediation of leukocyte migration across the endothelium [16,17]. This receptor also plays an important role in the inhibition of both systemic and tissue-specific inflammatory responses [18–20], and, more recently, has been implicated in both proatherosclerotic and atheroprotective effects, influencing the initiation and progression of atherosclerosis [21,22].

In platelets, we and others have shown that clustering or ligation of PECAM-1 inhibits signal transduction by the activatory collagen receptor GPVI, which hinders platelet aggregation and thrombus formation [8,9], although the mechanism for this inhibitory effect remains to be established. The inhibition of GPVI-stimulated platelet activation by PECAM-1 is associated with diminished protein tyrosine phosphorylation and decreased calcium mobilization [7]. We have found, however, that early tyrosine kinase-dependent signaling, including phosphorylation of the Fc receptor (FcR)γ-chain, spleen tyrosine kinase (Syk) and linker for activation of T cells (LAT), following stimulation of GPVI is largely unaffected by stimulation of PECAM-1 (data not shown). In this study, we therefore explored the next steps downstream, coordinated through the assembly of the LAT signalosome. Upon homophilic ligation and antibody-mediated clustering or following stimulation with collagen or thrombin, PECAM-1 becomes tyrosine-phosphorylated by Src-family kinases [6,23,24]. PECAM-1 also becomes tyrosine-phosphorylated following activation and aggregation of platelets, which is proposed to represent a negative feedback mechanism [6,7,23]. The cytoplasmic tail of human PECAM-1 has two distinct immunoreceptor tyrosine-based inhibitory motifs (ITIMs) surrounding tyrosines at positions 663 and 686 [25]. These ITIMs can serve as docking sites for signaling molecules such as non-receptor Src homology 2 (SH2) domain-containing protein tyrosine phosphatase-2 (SHP-2), which binds to the phosphorylated ITIMs through tandem SH2 domain-dependent interactions [26,27]. Several reports have strongly implicated SHP-2 in the functions of PECAM-1 in several cell systems [15,23,24,28,29].

SHP-2 is involved in the signaling pathways of a variety of growth factor-initiated and cytokine-initiated signal transduction processes, thereby regulating a range of cellular responses [30–33]. Although protein tyrosine phosphatases act to counter the effects of tyrosine kinase-dependent pathways, SHP-2, in most circumstances, plays a positive regulatory role in signal transduction, as previously reported for the regulation of growth factor receptor signaling [34]. Previous studies have demonstrated that a number of signaling proteins, such as Grb2, the p85 subunit of phosphatidylinositol 3-kinase (PI3K), and Grb2-associated binding protein 1 (Gab1), associate with SHP-2 after cytokine and growth factor receptor activation, leading to enhanced signal transduction [35]. Gab1 belongs to a family of scaffolding adaptor proteins, which have an N-terminal pleckstrin homology domain, multiple tyrosine-based motifs, and proline-rich sequences [36,37]. Upon growth factor, cytokine and antigen receptor stimulation, Gab1 provides a number of docking sites to mediate interactions with SH2 domain-containing proteins, such as SHP-2 and the p85 subunit of PI3K, mediating intracellular responses. Given the physiologic importance of the Gab1–SHP-2 association, it has been suggested that a primary role of Gab1 is to recruit SHP-2 [38]. Furthermore, SHP-2 regulates the amount of p85 that is bound to Gab1 by dephosphorylating p85-binding sites on Gab1 [38]. The physical association between p85 and Gab1 is important in mediating the PI3K signaling pathway induced by growth factors [37]. In this way, SHP-2 negatively regulates the Gab1–p85 interaction, controlling the kinetics and reducing the extent of PI3K signaling following epidermal growth factor stimulation [38].

LAT is an adaptor molecule that, upon phosphorylation by Syk, nucleates the formation of a protein complex that enables recruitment and activation of phospholipase C (PLC)γ2 following GPVI stimulation [39,40]. The activation of PLCγ2 in response to GPVI stimulation depends on recruitment of PI3K to phosphorylated LAT via the SH2 domains of the p85 subunit. Once recruited to the plasma membrane, PI3K phosphorylates phosphatidylinositol 4,5-bisphosphate to form phosphatidylinositol 3,4,5-trisphosphate [PtdIns(3,4,5)*P*_3_], to which multiple pleckstrin homology domain-containing proteins, such as PLCγ2 itself, Tec family kinases required for PLCγ2 activation, protein kinase B (PKB)/Akt and 3-phosphoinositide-dependent protein kinase 1 (PDK1), which phosphorylates and activates PKB/Akt, can bind, and become activated and mediate their functions [40,41].

In this study, we investigated the possibility that PECAM-1 regulates the formation of the LAT–Gab1–p85 signaling complexes and the potential effect of such interactions on GPVI-mediated platelet activation in platelets. We demonstrate that PECAM-1 interferes with the formation of Gab1–p85–SHP-2 complexes upon GPVI stimulation. These results provide a molecular explanation for PECAM-1-mediated inhibition of collagen-stimulated PI3K signaling, and thereby the inhibition of platelet function.

## Materials and methods

### Reagents

Anti-PECAM-1 monoclonal antibody for crosslinking (AB468) and an appropriate isotype control (AB600) were obtained from Autogen Bioclear (Nottingham, UK), and were dialyzed to remove azide. Goat anti-mouse IgG F(ab′)_2_ fragment antibody was obtained from Sigma Chemical (Poole, UK). Anti-PECAM-1 for immunoprecipitation (WM59) was obtained from Serotec (Oxford, UK). Anti-PECAM-1 for immunoblotting (C-20), anti-SHP-2 (C-18), anti-Gab1 (H-198) and protein A/G agarose were obtained from Santa Cruz (Autogen Bioclear, London, UK). Anti-PI3K p85 subunit (06-195) and anti-Akt/PKBα were obtained from Upstate Biotechnology (Dundee, UK). Anti-horseradish peroxidase (HRP)-conjugated secondary antibodies were obtained from New England Biolabs (Hitchin, UK), and enhanced chemiluminescence reagents were obtained from GE Healthcare (Chalfont St Giles, UK). Horm-Chemie collagen (collagen fibers from equine tendons) was obtained from Nycomed (Munich, Germany), and collagen-related peptide (CRP) was obtained from R. Farndale (University of Cambridge, UK). A plasmid containing cDNA encoding a glutathione-*S*-transferase (GST) fusion protein containing the N-terminal SH2 domain of p85 (GST–p85-N-SH2) was a gift from T. Pawson (University of Toronto, Ontario, Canada). PECAM-1 knockout mice were provided by T. Mak (University of Toronto, Ontario Canada). All protocols involving the use of animals were approved by the University of Reading Local Ethical Review Panel and authorized by a Home Office licence.

### Mouse platelet preparation and activation

Blood was obtained from PECAM-1 knockout and control mice via cardiac puncture after death. Blood (1 mL) was drawn into a syringe containing acidic citrate dextrose (100 μL;120 mm sodium citrate, 110 mm glucose, 80 mm citric acid) as anticoagulant. Platelets were prepared from whole blood by differential centrifugation in the presence of prostacyclin (0.1 μg mL^−1^), resuspended in modified Tyrode’s–HEPES buffer (134 mm NaCl, 0.34 mm Na_2_HPO_4_, 2.9 mm KCl, 12 mm NaHCO_3_, 20 mm HEPES, 5 mm glucose, 1 mm MgCl_2_, pH 7.3) to a density of 4 × 10^8^ cells mL^−1^, and rested for 30 min at 30 °C prior to experiments, as described previously [9,42,43]. For aggregation studies, platelets were suspended at a final concentration of 2.5 × 10^8^ cells mL^−1^, and aggregometry was performed at 37 °C in an optical platelet aggregometer (Chrono-log Corp., Havertown, PA, USA), as described previously [9].

Platelets from PECAM-1-deficient mice were found to have similar levels of LAT, Gab-1, p85, SHP-2 and PLCγ2 as platelets derived from wild-type mice (Fig. S1).

### Human platelet preparation and activation

Washed platelets were prepared from fresh blood obtained from aspirin-free donors by differential centrifugation, as described previously [44], and resuspended in modified Tyrode’s–HEPES buffer to a density of 4 × 10^8^ cells mL^−1^. Aggregation studies were performed at 37 °C in an optical platelet aggregometer (Chrono-log Corp.), as described previously [7]. For protein precipitation experiments, platelets were resuspended at 8 × 10^8^ cells mL^−1^ and rested for 30 min at 30 °C prior to experiments. PECAM-1 signaling was induced by antibody crosslinking with mouse monoclonal antibody AB468 (1 μg mL^−1^) and goat anti-mouse IgG (30 μg mL^−1^) for 5 min prior to agonist stimulation, as reported previously [7]. Mouse IgG antibody AB600 (1 μg mL^−1^) was used as the antibody control. Preincubation with IV.3 F(ab′) fragments, to block the low-affinity receptor for IgG FcγRIIA, did not alter the inhibitory effect of PECAM-1 crosslinking [45]. Stimulation of platelets with collagen (25 μg mL^−1^) or with crosslinking PECAM-1 antibodies in the presence of EGTA (1 mm) to prevent aggregation was performed at 37 °C in an optical platelet aggregometer (Chrono-log Corp.) with continuous stirring at 1200 r.p.m.. Informed consent from all human subjects donating blood was obtained, and procedures were approved by the University of Reading Research Ethics Committee.

### Immunoprecipitation and immunoblotting

For protein precipitation assays, platelets were suspended in buffer containing 1 mm EGTA, 10 μm indomethacin and 2 U mL^–1^ apyrase to prevent platelet aggregation, release of thromboxane A_2_, and the secondary effects of secreted ADP, respectively. Immunoprecipitation, sodium dodecylsulfate polyacrylamide gel electrophoresis (SDS-PAGE) and immunoblotting onto poly(vinylidine difluoride) (PVDF) membranes were performed with the use of standard techniques [7,43]. Normal IgG control was added to our immunoprecipitation experiments, and showed no effect on the interactions revealed in this study (Fig. S3). Quantification was performed following chemifluorescence detection with Typhoon Fluorescence Imaging software (GE Healthcare).

### Far-western blotting

GST–p85-N-SH2 was prepared as described previously [39]. SHP-2 immunoprecipitates from control or collagen-stimulated platelets were resolved by SDS-PAGE, transferred to PVDF membranes, blocked with bovine serum albumin protease-free solution, and incubated for 3 h with GST–p85-N-SH2 (10 μg mL^−1^), followed by anti-GST antibody (1 : 1000). Blots were washed and incubated for 2 h with HRP-conjugated anti-goat IgG antibody (1 : 8000), and signals were detected with a fluorescence imager (Typhoon; GE Healthcare).

### Statistical analysis

Determination of statistical significance was performed using Student’s paired *t*-test. Results are expressed as means ± standard errors of the mean.

## Results

### SHP-2 and p85 (PI3K) associate with PECAM-1 upon PECAM-1 or GPVI stimulation

**Fig. 1:**
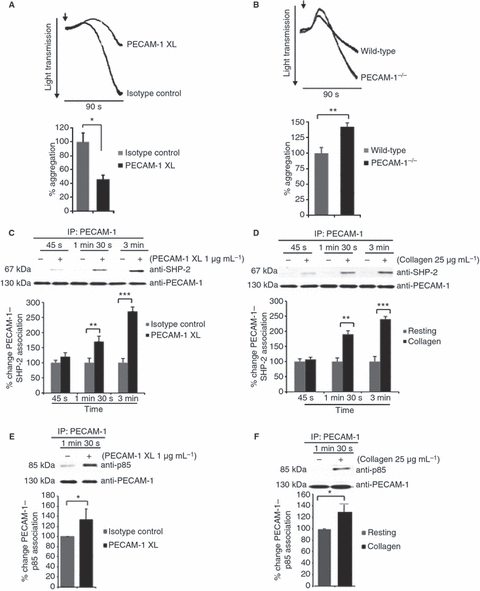
Stimulation of platelet endothelial cell adhesion molecule-1 (PECAM-1) signaling results in recruitment of phosphatidylinositol 3-kinase (PI3K). Washed human platelets were incubated with antibody specific for PECAM-1 crosslinking (XL) or isotype control prior to stimulation with collagen-related peptide (0.5 μg mL^−1^) for 90 s (A), or wild-type and PECAM-1-deficient mouse platelets were stimulated with collagen (2.5 μg mL^−1^) (B) and aggregation was measured under constant stirring conditions at 37 °C. Washed human platelets were treated with EGTA (1 mm), apyrase (2 U mL^−1^) and indomethacin (10 μm) prior to stimulation of PECAM-1 by antibody crosslinking (C, E) or with collagen (D, F) for 45, 90 and 180 s. (C, D) Levels of Src homology 2 domain-containing protein tyrosine phosphatase-2 (SHP-2) associated with PECAM-1 were detected before equivalent protein loading was verified by reprobing for PECAM-1. Levels of p85 subunit of PI3K associated with PECAM-1 detected after stimulation with glycoprotein VI agonist collagen (25 μg mL^−1^) (E) or antibody specific for PECAM-1 crosslinking (1 μg mL^−1^) (F). Equivalent protein loading was verified by reprobing for PECAM-1. Immunoblots were visualized by fluorescence imaging, quantified, and normalized for protein loading. Numerical data represent the percentage change of PECAM-1–SHP-2 association in stimulated samples as compared with control (mean ± standard error of the mean; *n* = 4). *t*-test: *P ≤ 0.05, **P ≤ 0.01, ***P ≤ 0.001. IP, immunoprecipitation.

PECAM-1 tyrosine phosphorylation and subsequent activation of signaling molecules is stimulated following PECAM-1 clustering (antibody or homophilic ligation) or following platelet activation [6,7]. Phosphorylation of PECAM-1 is associated with the inhibition of platelet function (Fig. 1A,B), as well as secretion and adhesion responses [6,7,9]. The activation of PECAM-1 signaling is also stimulated downstream of platelet activation, and has been proposed to represent a negative feedback mechanism [6,7]. The association of PECAM-1 with SHP-2 has been described previously, and shown to be mediated by the SH2 domains of this phosphatase [23,24,28,29]. In order to determine the kinetics and extent of SHP-2 recruitment by PECAM-1 following crosslinking of PECAM-1 or GPVI stimulation with collagen, human platelets were stimulated for 45 s, 1 min 30 s and 3 min, in the presence of EGTA (1 mm), apyrase (2 U mL^−1^) and indomethacin (10 μm) to prevent aggregation and ensure the study of primary signaling events. The level of SHP-2 associated with immunoprecipitated PECAM-1 was measured by immunoblot analysis. The extent of association between PECAM-1 and SHP-2 was dependent on the duration of stimulation, and was proportional to the increase in the level of tyrosine phophorylation of SHP-2 (Fig. 1C,D; Fig. S2). Changes in tyrosine phosphorylation, SHP-2 binding and PECAM-1 binding were detected at early time points (detectable at 45 s in Fig. S2) and continued to rise for 3 min. Under the conditions used, similar kinetics for PECAM-1–SHP-2– interactions were observed for PECAM-1 crosslinking and stimulation of platelets with collagen. In subsequent experiments, a time point of 90 s was chosen to ensure that quantification of association could be reliably measured with this approach.

To explore the possibility that components of the activatory GPVI pathway interact with PECAM-1, we investigated the potential association between the p85 subunit of PI3K and PECAM-1 following PECAM-1 or GPVI stimulation. Human platelets were incubated with or without crosslinking with an antibody specific for PECAM-1 for 90 s, as described in Materials and methods. The p85 subunit of PI3K was found to associate with PECAM-1, and the level of this association was increased significantly upon stimulation of either PECAM-1 or GPVI signaling (Fig. 1E,F).

### p85 associates with SHP-2 upon PECAM-1 crosslinking or GPVI stimulation

**Fig. 2:**
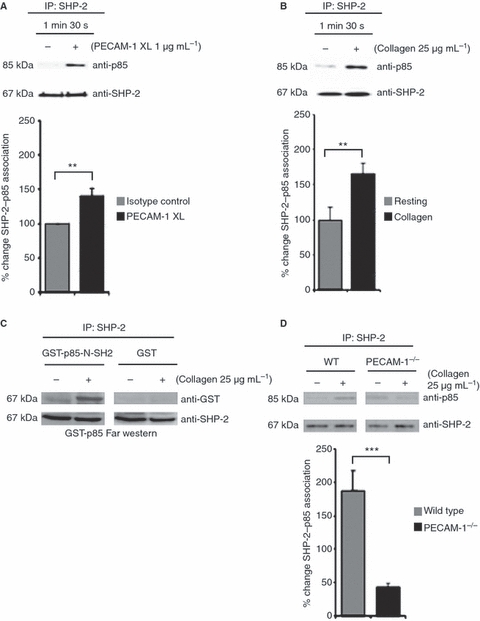
Platelet endothelial cell adhesion molecule-1 (PECAM-1) regulates the association of p85 with SHP-2. Washed human platelets (A, B) and platelets derived from PECAM-1-deficient and wild-type (WT) mice (D) were treated with EGTA (1 mm), apyrase (2 U mL^−1^) and indomethacin (10 μm) prior to PECAM-1 stimulation by antibody crosslinking (XL) (A) or stimulation with collagen for 90 s (B, D). The levels of p85 associated with SHP-2 were detected before equivalent protein loading was verified by reprobing for Src homology 2 domain-containing protein tyrosine phosphatase-2 (SHP-2). (C) Far-western blotting for SHP-2–p85 interaction was performed on lysates of cells stimulated with collagen (25 μg mL^−1^) for 90 s, resolved by sodium dodecylsulfate polyacrylamide gel electrophoresis and transferred to poly(vinylidine difluoride) membranes. The membranes were incubated with glutathione-*S*-transferase (GST) fusion protein containing the N-terminal SH2 domain of p85 (GST–p85-N-SH2) or GST control, followed by anti-GST and secondary antibodies. Blots were washed and incubated for 2 h with horseradish peroxidase-conjugated anti-goat IgG antibody (1 : 8000), and signals were detected by chemifluorescence. Numerical data represent the percentage change of SHP-2–p85 association in stimulated samples as compared with the control (mean ± standard error of the mean; *n* = 4). *t*-test: **P ≤ 0.01 and ***P ≤ 0.001. IP, immunoprecipitation.

Previous studies in other cell models have suggested that the SH2 domains of p85 direct the interaction of the PI3K complex with activated growth factor receptors and signaling intermediate molecules such SHP-2, Gab1, Grb-2-associated binding protein-2, Grb2, and SHIP [38]. Given the role of PECAM-1 in the negative regulation of platelet function and the recruitment of SHP-2 to this ITIM-containing receptor, we investigated whether the p85 subunit of PI3K associates with SHP-2 upon PECAM-1 crosslinking or GPVI stimulation. As shown in Fig. 2A,B, SHP-2 was immunoprecipitated from the lysates of resting platelets and following stimulation of PECAM-1 and GPVI signaling. Low levels of p85 were found to be present in SHP-2 immunoprecipitates from unstimulated platelets, and this association was increased notably following stimulation of PECAM-1 or activation of platelets with collagen. In order to explore a potential direct interaction between SHP-2 and the p85 subunit of PI3K, we used GST–p85-N-SH2 in far-western blots. Resting and collagen-stimulated samples were lysed, and SHP-2 was immunoprecipitated. Immunoprecipitates were separated by SDS-PAGE and transferred to PVDF membranes. After incubation with GST–p85-N-SH2 or GST alone (control), the presence of bound fusion protein was detected with an anti-GST antibody and chemifluorescence detection. An increase in GST–p85-N-SH2 binding to immunoprecipitated SHP-2 following GPVI stimulation (Fig. 2C) suggested that the p85 subunit of PI3K is capable of binding directly to SHP-2.

### PECAM-1 modulates SHP-2–p85 association

As SHP-2 is capable of binding p85 directly, it is possible that PECAM-1 (or binding of PECAM-1 to SHP-2) drives this association. We therefore evaluated the interaction between p85 and SHP-2 in whole platelet lysates from control (wild-type) and PECAM-1-deficient platelets stimulated with collagen. Substantially lower levels of collagen-stimulated SHP-2–p85 association were detected in PECAM-1-deficient platelets than in control platelets (Fig. 2D). These data strongly indicate that PECAM-1 modulates SHP-2–p85 association.

### PECAM-1 signaling destabilizes a collagen-stimulated Gab1–p85 complex

**Fig. 3:**
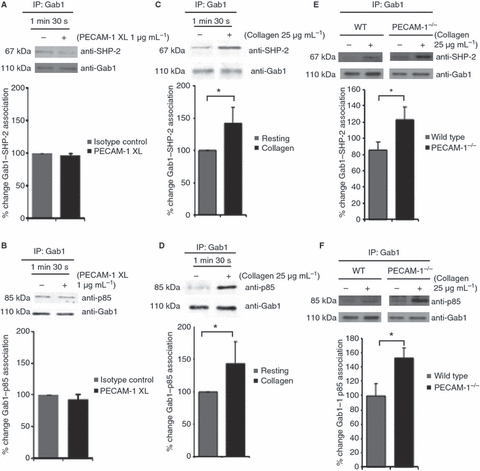
The adaptor protein Grb-2-associated binding protein-1 (Gab1) associates with Src homology 2 domain-containing protein tyrosine phosphatase-2 (SHP-2) and phosphatidylinositol 3-kinase on platelet activation. These associations are enhanced in the absence of platelet endothelial cell adhesion molecule-1 (PECAM-1). Gab1 was immunoprecipitated from washed human platelets and platelets derived from PECAM-1-deficient and wild-type (WT) mice following stimulation of PECAM-1 signaling by antibody crosslinking (XL) (A, B) or collagen (C–F) for 90 s. Proteins were separated by sodium dodecylsulfate polyacrylamide gel electrophoresis and immunoblotted to detect SHP-2 (A, C, E) and p85 (B, D, F). Numerical data represent the percentage change of Gab1–SHP-2 or Gab1–p85 association in stimulated samples as compared with the control (mean ± standard error of the mean; *n* = 4). *t*-test: *P ≤ 0.05. IP, immunoprecipitation.

In different cell types, Gab1 has been shown to contain a number of different docking sites that mediate independent interactions with SH2 domain-containing proteins such as SHP-2 and the p85 subunit of PI3K. The formation of these complexes is involved in signaling events mediated by cytokine and tyrosine kinase receptors [36,37]. Given our finding that, in human platelets, SHP-2 interacts directly with p85 in a manner that depends on the presence of PECAM-1, we hypothesized that PECAM-1 may bind to SHP-2–p85 complexes and interfere with the ability of either of these molecules to bind to Gab1. To test this hypothesis, we investigated the effect of PECAM-1 crosslinking or PECAM-1 deficiency on the ability of SHP-2 and p85 to interact with Gab1 in GPVI-activated platelets. PECAM-1 crosslinking had no effect on the levels of association of either SHP-2 (Fig. 3A) or p85 (Fig. 3B) with Gab1 in unstimulated platelets. We found that the levels of association of SHP-2 and p85 with Gab1 in Gab1 immunoprecipitates, which are low in resting human platelets, increased upon stimulation of platelets with collagen (Fig. 3C,D). Gab1–SHP-2 interactions were also found to be increased in SHP-2 immunoprecipitates (Fig. S3F). The effect of PECAM-1 on levels of association of p85 or SHP-2 with Gab1 was investigated with mouse platelets deficient in PECAM-1. Significantly higher levels of association of SHP-2 (Fig. 3E) or p85 (Fig. 3F) with Gab1 were observed in collagen-stimulated platelets derived from PECAM-1-deficient mice than in those from wild-type mice. On the basis of these findings, we conclude that PECAM-1 competes with Gab1 for association with SHP-2 in GPVI-stimulated platelets. Furthermore, the ability of PECAM-1-associated SHP-2 to complex with the p85 subunit of PI3K limits the amount of p85 available to bind to Gab1 downstream of GPVI stimulation.

### LAT-mediated PI3K signaling is modulated by PECAM-1

Upon GPVI stimulation, LAT forms a platform for the assembly of a signaling complex that includes PI3K and other downstream molecules, which results in the activation of PI3K signaling [39,40,46].

**Fig. 4:**
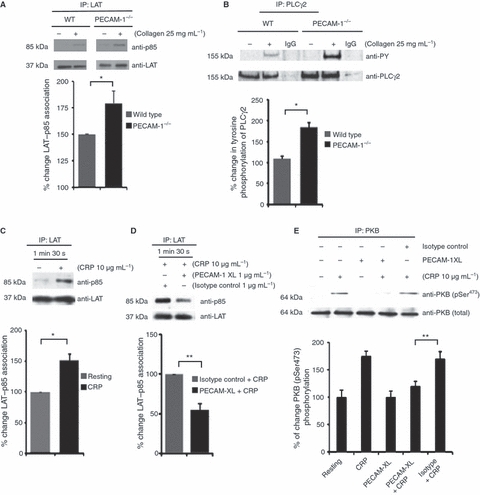
The linker for activation of T cells (LAT) signalosome is modulated by platelet endothelial cell adhesion molecule-1 (PECAM-1). Washed human platelets and platelets derived from PECAM-1-deficient and wild-type (WT) mice were treated with EGTA (1 mm), apyrase (2 U mL^−1^) and indomethacin (10 μm) prior to stimulation with collagen or collagen-related peptide (CRP) for 90 s, and for human platelets in the presence or absence of PECAM-1 activation by antibody crosslinking (XL). (A, C, D) Levels of p85 associated with LAT were detected before equivalent protein loading was verified by reprobing for LAT. (B) Levels of phospholipase C (PLC)γ2 phosphorylation were determined before equivalent protein loading was verified by reprobing for PLCγ2. (E) Human platelets were stimulated with the glycoprotein VI-specific agonist CRP, and the effects of prior stimulation of PECAM-1 signaling, through antibody-mediated crosslinking (PECAM-1 XL), was determined by Western blot analysis of whole cell extracts separated by sodium dodecylsulfate polyacrylamide gel electrophoresis. Phosphatidylinositol 3-kinase signaling was measured through assessment of protein kinase B (PKB)α/Akt phosphorylation (Ser473) by immunoblot analysis with a phosphospecific antibody. Equivalent protein loading was verified by reprobing for total PKB/Akt. Densitometry analysis was performed on replicate experiments, and data were normalized for total protein loading levels (mean ± standard error of the mean; *n* = 4). *t*-test: *P ≤ 0.05 and **P ≤ 0.01.

On the basis of our finding that PECAM-1–SHP-2–p85 complex formation limits the amount of p85 available to Gab1 in GPVI-stimulated platelets, we hypothesized that PECAM-1 would also affect the assembly of the LAT signalosome. To test this hypothesis, the effect of PECAM-1 on interactions between LAT and p85 was investigated in control and PECAM-1-deficient mouse platelets following stimulation with collagen. We found that the absence of PECAM-1 was associated with a significant increase in the levels of interaction between LAT and p85 (Fig. 4A). Consistent with this and increased levels of PI3K signaling in the absence of PECAM-1, collagen-stimulated PLCγ2 tyrosine phosphorylation was also found to be increased (Fig. 4B). These results indicate that PECAM-1 modulates the assembly of the LAT signalosome, which is consistent with the regulation of PI3K signaling leading to reductions in the PLCγ2 functions of calcium regulation and α-granule secretion [6,7,45]. To further substantiate this model, and also in human platelets, the effect of stimulation of PECAM-1 on GPVI-mediated recruitment of PI3K to LAT was tested. For these experiments, the GPVI-specific agonist CRP was used, because, with the combinations of antibodies present, this allowed reliable quantification. The levels of LAT-associated p85 were determined in resting and collagen-stimulated human platelets. As shown in Fig. 4C, low levels of p85 were found to be present in LAT immunoprecipitates from resting platelets, and this association was increased significantly following stimulation of platelets with CRP. In order to explore whether this association would be affected by PECAM-1 downstream signaling on GPVI signaling, levels of LAT-associated p85 were determined upon stimulation of PECAM-1 following GPVI-mediated activation with CRP. The levels of p85 associated with LAT decreased significantly when PECAM-1 was stimulated by crosslinking prior to CRP stimulation (Fig. 4D). To confirm that this resulted in diminished PI3K signaling, we investigated the effect of PECAM-1 crosslinking on PKB/Akt activation, which is a downstream consequence of recruitment of PI3K to LAT, in GPVI-activated human platelets. PKB/Akt is activated by PDK1-mediated phosphorylation of Ser473; therefore, PKB/Akt activation was measured by immunoblot analysis with an antibody specific for the phosphorylated form of Ser473 (pSer473). We found that PECAM-1 crosslinking resulted in inhibition of GPVI-stimulated PKB/Akt Ser473 phosphorylation (Fig. 4E). PECAM-1 crosslinking antibody alone did not affect PKB/Akt phosphorylation. These results indicate that collagen-stimulated PI3K activation, which is dependent on recruitment of p85 to LAT in response to GPVI signaling, is negatively regulated by PECAM-1 in human platelets. On the basis of these results, we propose that PECAM-1-mediated inhibition of GPVI-dependent platelet responses results, at least in part, from recruitment of SHP-2–p85 complexes to tyrosine-phosphorylated PECAM-1, which destabilizes the PI3K association with the activatory signaling molecules Gab1 and LAT.

## Discussion

A number of recent studies have shown that the scaffolding adaptor protein Gab1 is critical for signaling by a number of receptor tyrosine kinases, cytokines, and antigen receptors [38]. Tyrosine-phosphorylated Gab1 provides docking sites for multiple SH2 domain-containing signaling molecules, such as SHP-2, the p85 regulatory subunit of PI3K, Crk, and PLCγ, which transduce signals following cytokine receptor stimulation [37]. One of these binding partners, SHP-2, which is able to dephosphorylate a number of signaling molecules [47], has been shown to interact with Gab1, causing dephosphorylation of Gab1-associated phosphoproteins [47]. In platelets, it has been found that Gab1 is associated with SHP-2 and p85 in response to thrombopoietin [48], and one possible explanation for the role of the association of SHP-2 with Gab1 is that this association may influence the interaction between Gab1 and the p85 subunit of PI3K, therefore affecting downstream signaling.

**Fig. 5:**
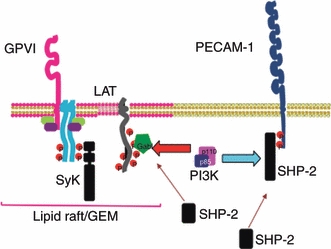
Working model for the modulation of collagen-stimulated phosphatidylinositol 3-kinase (PI3K) signaling and platelet function by platelet endothelial cell adhesion molecule-1 (PECAM-1). Homophilic ligand binding or clustering of PECAM-1 or glycoprotein (GP)VI activation by collagen results in stimulation of tyrosine phosphorylation of the immunoreceptor tyrosine-based inhibitory motifs present in the cytoplasmic tail of PECAM-1. This results in the recruitment and activation of the tyrosine phosphatase Src homology 2 domain-containing protein tyrosine phosphatase-2 (SHP-2). Collagen stimulation of platelets results in the formation of a complex between PI3K and the adaptor protein Grb-2-associated binding protein-1 (Gab1), which also binds to linker for activation of T cells (LAT), forming a signaling complex. SHP-2 is also capable of joining this complex, an interaction that is enhanced in the absence of PECAM-1 signaling. The stimulation of PECAM-1 results in the recruitment of p85 to bind to PECAM-1. The ability *in vitro* of SHP-2 to directly interact with p85 suggests that the interaction of p85 and PECAM-1 is mediated indirectly by the phosphatase. Indeed, the interaction between SHP-2 and p85 is dramatically reduced in the absence of PECAM-1, suggesting that PECAM-1 controls this interaction. Consistent with studies in another cell types where SHP-2 disrupts Gab1 and p85 interactions, through dephosphorylation of a tyrosine required for binding, the absence of PECAM-1 results in stabilization of the interaction between Gab1 and p85. This indicates that PECAM-1 signaling results in the loss of PI3K from the LAT signalosome and reduced levels of PI3K signaling. The relative redistribution of p85 from the LAT signalosome may be correlated with the inhibition of PI3K signaling. This provides a mechanism by which the activation of PECAM-1 results in negative feedback to activation pathways. GEM, glycolipid-enriched membrane; Syk, spleen tyrosine kinase.

Our working model (Fig. 5) shows that the activation of platelets results in PECAM-1 phosphorylation and signaling, providing negative feedback to activation pathways. Collagen stimulation of platelets results in the formation of a complex between PI3K and the adaptor protein Gab1, which also binds to LAT, forming a signaling complex. Gab1 also interacts with SHP-2, another component capable of joining this complex, in collagen-stimulated platelets, and this interaction is enhanced in the absence of PECAM-1 signaling. The stimulation of PECAM-1 results in the recruitment of p85 to PECAM-1, and enhances the ability of SHP-2 to interact with p85. The ability *in vitro* of SHP-2 to directly interact with p85 supports the notion that the interaction of p85 with PECAM-1 is mediated indirectly by the phosphatase. Furthermore, the substantial reduction in the interaction between SHP-2 and p85 in the absence of PECAM-1 suggests that PECAM-1 controls this association. Consistent with what has been found for other cell types [38], our model highlights the ability of PECAM-1 to modulate the assembly of the LAT signalosome, where PECAM-1 activation and SHP-2 recruitment result in diminished association of the p85 subunit of PI3K with Gab1 and LAT, moving p85 from a substrate-rich to a substrate-poor environment (80% of PECAM-1 is excluded from lipid rafts) [49]. This would lead to a redistribution of p85 from LAT-containing lipid raft compartments to PECAM-1 signaling complexes, causing a reduction in collagen-mediated signaling through relocation of the enzyme away from the activated collagen receptor complex.

In platelet activation, LAT forms a platform for the assembly of a signaling complex that includes PLCγ2, which in turn becomes tyrosine-phosphorylated. PI3K is also recruited and, through the generation of PtdIns(3,4,5)*P*_3_, influences the recruitment and activation of PLCγ2, which liberates the second messengers 1,2-diacylglycerol and inositol 1,4,5-trisphosphate [39,40]. The formation of these molecules is responsible for the mobilization of calcium from intracellular stores and activation of isoforms of protein kinase C, leading to secretion and aggregation. PI3K activity also results in the regulation of PKB, which is important for platelet function and thrombus formation [39,41]. We recently demonstrated that PECAM-1 signaling is capable of inhibiting activatory signaling stimulated by ADP and thrombin [45], suggesting that PECAM-1 may control a broad inhibitory mechanism in these cells. This potential has been also reported for another platelet ITIM receptor, G6B [50]. As LAT and its role in platelet signaling is restricted to ITAM receptors, it is not yet fully understood how PECAM-1 may inhibit signaling stimulated by ADP and thrombin. One possible explanation is that calcium mobilization following stimulation of platelets is diminished through PECAM-1 signaling [7], indicating that modulation of PI3K and PLCγ2 may also underlie inhibition in this context. Given the ability of PECAM-1 to modulate signaling protein complex formation (e.g. LAT–Gab1–p85 and SHP-2–p85) following collagen stimulation, the potential role of PECAM-1 in regulating isoforms of PI3K that are involved in GPVI-mediated and non-GPVI-mediated platelet activation, such as p110β [51,52], which couples to the p85 regulatory subunit, will be a focus of future investigations.

Our working model suggests that the relative redistribution of p85 from lipid raft compartments may be correlated with the inhibition of PI3K signaling and downstream effects such as the inhibition of calcium mobilization, as we have previously described [7]. This may represent a competitive relationship between the LAT and PECAM-1 signalosomes, providing a balance between ITAM-containing and ITIM-containing receptors when they are required on the same cell. Further work is required to understand the kinetics and activation of these and other molecules involved in this complex process.

Our findings indicate that PECAM-1, through regulation of protein complex formation, modulates the subcellular localization of PI3K, thereby diminishing GPVI-stimulated PI3K signaling.

## Supporting Information

**Fig. S1.** Levels of SHP-2, p85, LAT, Gab-1, PLCγ2 and PECAM-1 derived from whole cell lysates of platelets from wild-type and PECAM-1-deficient mice.

**Fig. S2.** Levels of tyrosine phosphorylation of SHP-2 upon GPVI stimulation.

**Fig. S3.** Modulation of collagen-stimulated p85 interactions by PECAM-1.
