# Supplementary material for: Platelet endothelial cell adhesion molecule-1 regulates collagen-stimulated platelet function by modulating the association of phosphatidylinositol 3-kinase with Grb-2-associated binding protein-1 and linker for activation of T cells
Source: J Thromb Haemost. 2010 Nov;8(11):2530–41. doi: 10.1111/j.1538-7836.2010.04025.x (PMC3298659; doi:10.1111/j.1538-7836.2010.04025.x)
Supplement: Supplementary file 1 [file jth0008-2530-SD1.doc]

**Figure 1** – Levels of SHP-2, p85, LAT, GAB-1, PLCγ2 and PECAM-1 derived from wild-type and PECAM-1 deficient platelets mice whole cell lysates.

**Figure 2 – Levels of tyrosine phosphorylation of SHP-2 upon GPVI stimulation.** Washed human platelets were treated with EGTA (1 mM), apyrase (2 U /mL^-1^) and indomethacin (10 µM) prior to stimulation with collagen for 45, 90 and 180 s. SHP-2 was immunoprecipitated and immunoblotted to detect phosphotyrosine residues. Equivalent protein loading was verified by reprobing for SHP-2.

**Figure 3 – Modulation of collagen-stimulated p85 interactions by PECAM-1.** Washed human platelets and platelets derived from PECAM-1 deficient and wild-type mice were treated with EGTA (1 mM), apyrase (2 U /mL^-1^) and indomethacin (10 µM) prior to stimulation with collagen or PECAM-1 cross-linking. Normal IgG control was added in our immunoprecipitation experiments demonstrating no effect in the interactions showed in this study. Equivalent protein loading was verified by reprobing to the specific antibodies. (anti-PECAM-1 (a), anti-SHP-2 (b,c), anti Gab-1 (d) and anti-LAT (e). **Reverse co-imunoprecipitation** of Gab1-SHP-2 association with Normal IgG control (f).
